# Unveiling multiscale spatiotemporal dynamics of volatility in high-frequency financial markets

**DOI:** 10.1371/journal.pone.0315308

**Published:** 2024-12-30

**Authors:** Fangyan Ouyang, Wenyan Peng, Tingting Chen

**Affiliations:** 1 School of Media Engineering, Communication University of Zhejiang, Hangzhou, China; 2 School of Mathematics and Statistics, Hunan University of Technology and Business, Changsha, China; 3 Key Laboratory of Hunan Province for Statistical Learning and Intelligent Computation, Hunan University of Technology and Business, Changsha, China; 4 Department of Finance, Zhejiang University of Finance and Economics, Hangzhou, China; University of Almeria: Universidad de Almeria, SPAIN

## Abstract

This study explores the intricate dynamics of volatility within high-frequency financial markets, focusing on 225 of Chinese listed companies from 2016 to 2023. Utilizing 5-minute high-frequency data, we analyze the realized volatility of individual stocks across six distinct time scales: 5-minute, 10-minute, 30-minute, 1-hour, 2-hour, and 4-hour intervals. Our investigation reveals a consistent power law decay in the auto-correlation function of realized volatility across all time scales. After constructing cross-correlation matrices for each time scale, we analyze the eigenvalues, eigenvectors, and probability distribution of *C*_*ij*_ based on Random Matrix Theory. Notably, we find stronger correlations between stocks at higher frequencies, with distinct eigenvector patterns associated with large eigenvalues across different time scales. Employing Planar Maximally Filtered Graphs method, we uncover evolving community structures across the six time scales. Finally, we explore reaction speed across multiple time scales following big events and compare industry-specific reactions. Our findings underscore the faster reaction speed at higher frequency scales, shedding light on the multifaceted dynamics of high-frequency financial markets.

## Introduction

In modern financial markets, high-frequency trading (HFT) has become an essential aspect. Advances in technology enable HFT participants to execute a large number of trades in very short periods, significantly affecting market volatility. Studying the volatility in high-frequency trading is crucial for understanding market behavior, predicting price changes, and developing investment strategies.

In the current technological era, big data is a critical issue in both business and technology domains [1]. The accumulation of extensive historical financial data in stock markets enables the exploration of the fine structure of financial dynamics, leading to various empirical findings [2–10]. It can also provide invaluable insights into market dynamics, risk management, and investment options. As financial markets grow more complex, data analysis has shifted from simple reporting to a strategic tool for gaining a competitive edge [11, 12]. It is crucial in multiple areas of finance management, such as risk assessment, portfolio management, fraud detection, and strategic planning [12]. With the rise of online big data, new methods have emerged, enhancing results. For instance, price changes can be predicted using collective mood states from Twitter [13], and trading behavior can be quantified with Google Trends and Wikipedia view times [14, 15]. Big data is also a significant factor in business process management and HR processes, supporting decision-making [16].

High-frequency data provides more detailed insights into the tail dependence between the financial system and its institutions than daily returns. Previous studies have mostly relied on low-frequency data with sampling frequencies of day, week, month, quarter, or year, which fail to accurately capture intra-day volatility [17]. High-frequency data, sampled at hours, minutes, or even shorter intervals, contains rich information about asset prices. With advancements in information technology, accessing high-frequency data has become faster and cheaper. It is an opportune time to use high-frequency data to explore the intrinsic mechanisms of the price movements for each stock, and to obtain more information about stock prices.

Recent unexpected changes in macroeconomic conditions, international events, and economic policies have increased financial market volatility [18]. Some research has detected jump volatility in financial assets using high-frequency data [19]. Jump volatility, which represents infrequent but sharp changes in asset prices, describes market volatility more accurately than continuous volatility [20]. For instance, Wright and Zhou (2007) found that jump volatility explains much of the counter-cyclical movements in bond risk premiums [21]. Zhang et al. (2016) identified jump volatility as a significant component of the Dow Jones Industrial Average stocks’ volatility [22], and Audrino and Hu (2016) showed that it improves the forecast of the S&P 500’s volatility [23]. Despite the extensive research on financial market volatility, most studies rely on low-frequency data, which cannot accurately reflect intra-day volatility information. Despite the insights provided by jump volatility, realized volatility, which captures both continuous price movements and jumps by summing squared high-frequency returns, offers a more comprehensive measure of market variability. Andersen et al. (2012) introduced jump-robust estimators that enhance realized volatility measurement by mitigating the impact of jumps [24]. Nevertheless, most studies rely on low-frequency data, which cannot accurately reflect intra-day volatility information.

Effective policy making and regulation in financial markets depend on a deep understanding of the complexity. Network analysis is an innovative method that enhances data mining and knowledge discovery in financial data. Based on complex network theory, the topological structures of a market can be extracted to uncover hidden information and relationships among stocks [25]. Academically, network analysis can identify new features and dynamics of international trade, both wholly and partially [26]. Complex network theory is widely applied to analyze the topological characteristics, time evolution, community evolution, and competition patterns of mineral resource trade networks, such as coal [27], fossil fuels [28], boron ore [29], lithium [30], nickel [31], and barite [32]. Additionally, previous research found that eigenmodes for large eigenvalues are often dominated by a community of stocks associated with a specific business sector based on Random Matrix Theory (RMT) [33]. Planar Maximally Filtered Graphs (PMFG) method has also been introduced to uncover community structure [34].

Motivated by the above discussion, in this paper, we construct realized volatility spillover networks by employing the 5-min high frequency data of the Chinese listed companies. Based on RMT, we analyze the statistical properties of the eigenvalues and eigenvectors of the correlation matrix. With the the PMFG method [5, 8, 35–37], the community structures across six time scales are analyzed. To explore the reaction of the stocks and industries to the big events happened in financial markets, we focus on two typical big events in China’s stock markets, one in 2021 and another in 2022. The term ‘Big Events’ refers to environmental, economic, and other major disruptions that cause social instability [38]. Examples include natural disasters, political transitions, and economic recessions. These big events significantly affect the stock markets, and draw the attention of the scientists in various fields. Investigating the effects of these big events is important and necessary.

The remainder of the paper proceeds as follows. In Section 2, we describe the data and methodology. In section 3, we present the results and our main findings. Section 4 is conclusions and some discussions.

## Materials and methods

### Data description

Our study involves 5-min high-frequency price data on the constituent stocks of Shanghai and Shenzhen stock markets. The data, spanning seven years (2016 to 2023), is freely accessible from https://www.joinquant.com. The collection and analysis methods adhered to the terms and conditions specified by the data source. Specifically, we collected 5-min data of 225 stocks with the time period from 9:35 of 2016.1.4 to 15:00 of 2023.6.29. The time length for each stock is 87360 data points.

### Calculation of the realized volatility

To analyze this data, we first calculate the realized volatility for each stock. This calculation is essential for understanding the immediate price movements and is performed for six distinct time scales.

Here, we use *P*_*i*_(*t*′) to denote the price of the *i*-th stock at time *t*′. To avoid long-term trends, we define the logarithmic price return as

$$\begin{array}{c} R_{i}\left(t^{\prime },\Delta t\right)=\text{ln}\left[P_{i}\left(t^{\prime },\Delta t\right)/P_{i}\left(t^{\prime }-1,\Delta t\right)\right], \end{array}$$ (1)

where Δ*t* = 1, 2, 6, 12, 24, 48.

Then, we employ the estimated realized volatility *RV* introduced by Andersen et al. [24].

$$\begin{array}{c} RV_{i}\left(t^{\prime },\Delta t\right)=\sum_{t^{\prime }=1}^{M}R_{i}{\left(t^{\prime },\Delta t\right)}^{2}, \end{array}$$ (2)

where *M* = 48, 24, 8, 4, 2, 1, and *i* corresponds to the *i*-th stock. By capturing both continuous price movements and discrete jumps, the estimated realized volatility offers a comprehensive measure of market variability. Furthermore, realized volatility can be directly computed from high-frequency data, facilitating a more precise and timely evaluation of market conditions.

Then we introduce the normalized realized volatility

$$\begin{array}{c} rv_{i}\left(t^{\prime },\Delta t\right)=\left[RV_{i}\left(t^{\prime },\Delta t\right)-\left\langle RV_{i}\left(t^{\prime },\Delta t\right)\right\rangle \right]/\sigma_{i}, \end{array}$$ (3)

where 〈⋯〉 represents the time average over time *t*′, and $\sigma_{i}=\sqrt{\langle RV_{i}{\left(t^{\prime },\Delta t\right)}^{2}\rangle -{\langle RV_{i}\left(t^{\prime },\Delta t\right)\rangle }^{2}}$ is the standard deviation of *RV*_*i*_(*t*′, Δ*t*) [8].

Afterwards, the auto-correlation function for each time scale is calculated. The auto-correlation function of volatilities measures the persistence and temporal dependencies in market volatility, which is crucial for accurate volatility forecasting. The auto-correlation function of volatilities of the *i*-th stock for each time scale is defined as

$$\begin{array}{c} A_{i}\left(t,\Delta t\right)=[\langle |rv_{i}\left(t^{\prime },\Delta t\right)||rv_{i}\left(t^{\prime }+t,\Delta t\right)\left|\right\rangle -\langle |rv_{i}\left(t^{\prime },\Delta t\right){\left|\right\rangle }^{2}]/A_{i}\left(0,\Delta t\right), \end{array}$$ (4)

with *A*_*i*_(0, Δ*t*) = 〈|*rv*_*i*_(*t*′, Δ*t*)|^2^〉 − 〈|*rv*_*i*_(*t*′, Δ*t*)|〉^2^. It is well known that the volatility in financial dynamics is long-range correlated in time, i.e., *A*(*t*) decays by a power law [3, 4, 39, 40].

### Construction and analysis of the cross-correlation matrix

The cross-correlation matrix helps in identifying the relationships between different stocks, providing insights into how price movements are interlinked within the market. In this part, we construct the cross-correlation matrix for each time scale.

The elements of the equal-time cross-correlation matrix *C* for the 225 stocks for time scale Δ*t* are defined by

$$\begin{array}{c} C_{ij}\left(\Delta t\right)=\left\langle rv_{i}\left(t^{\prime },\Delta t\right)rv_{j}\left(t^{\prime },\Delta t\right)\right\rangle , \end{array}$$ (5)

which measures the correlations between the returns of individual stocks. According to the definition, *C*(Δ*t*) is a real symmetric matrix, and the value of *C*_*ij*_(Δ*t*) ranges from −1 to 1. Following this method, the equal-time cross-correlation matrix *C*(Δ*t*) for different time scales is constructed.

The reliability of financial correlation matrices is significantly impacted by noise, especially in large datasets. Employing the RMT theory has become a critical methodology in financial data analysis, as it effectively filters out noise, thereby providing more accurate and insightful representations of the true underlying structure of financial markets [34].

The Wishart matrix is derived from non-correlated time series. Assuming that there are *N* time series with a length *T*, statistical properties of such random matrices are well understood [41, 42]. In the limit *N* → ∞ and *T* → ∞ with *Q* ≡ *T*/*N* ≥ 1, the probability distribution *P*_*rm*_(λ) of the eigenvalue λ is given by [41, 42]

$$\begin{array}{c} P_{rm}\left(\lambda \right)=\frac{Q}{2\pi }\frac{\sqrt{\left(\lambda_{max}^{ran}-\lambda \right)\left(\lambda -\lambda_{min}^{ran}\right)}}{\lambda }, \end{array}$$ (6)

and the lower and upper bounds of λ are

$$\begin{array}{c} \lambda_{min(max)}^{ran}={\left[1\pm (1/\sqrt{Q})\right]}^{2}. \end{array}$$ (7)

For a real dynamic system, large eigenvalues deviating from *P*_*rm*_(λ) of the Wishart matrix imply that there exist non-random interactions. Both mature and emerging stock markets show such a phenomenon [5]. In our notations, the eigenvalues are arranged in the order of λ_*α*_ > λ_*α*+1_, with *α* = 0, …, *N* − 1, with *N* being the number of stocks.

Based on the RMT theory, the cross-correlation between two stocks can be decomposed into different eigenmodes [43],

$$\begin{array}{c} C_{ij}=\sum_{\alpha =1}^{N}\lambda_{\alpha }C_{ij}^{\alpha },\;C_{ij}^{\alpha }=V_{\alpha ,i}V_{\alpha ,j}, \end{array}$$ (8)

where *V*_*α*,*i*_ is the *i*-th component in the eigenvector of λ_*α*_, and $C_{ij}^{\alpha }$ represents the cross-correlation in the *α*-th eigenmode, sorted by eigenvalue from largest to smallest.

Previous research has found that the eigenmodes for the large eigenvalues are dominated by a community of stocks, usually associated with a business sector. Inspired by the work in ref. [43], a methodology based on RMT is introduced. The largest eigenvalue λ_0_ represents the market mode, which is driven by interactions common for stocks in the entire market. Other large eigenvalues usually correspond to the business sectors.

Building on this foundation, the identified modes associated with large eigenvalues illustrate how stocks behave collectively and relate to specific business sectors. These modes reveal common patterns in price movements and volatility dynamics, indicating that stocks within the same sector or across different sectors respond similarly to market fluctuations. Understanding these modes helps assess sectoral interactions and informs strategies for portfolio diversification and risk management.

## Results

### Time scale analysis of realized volatility auto-correlation

In this section, we analyze the auto-correlation function of realized volatility across different time scales. This analysis helps us understand the persistence and decay patterns of volatility over time, providing insights into the temporal dynamics of financial markets. By examining the auto-correlation function, we can identify how quickly information is assimilated into prices and how past volatility influences future volatility.

**Fig 1:**
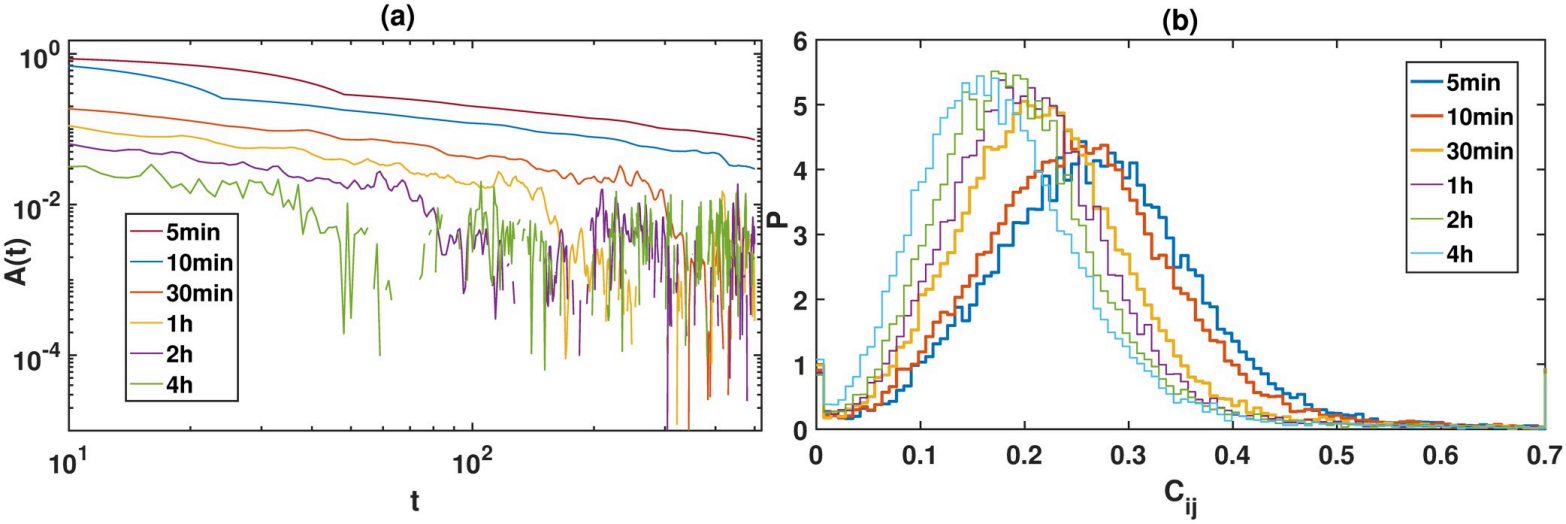
Sub-figure (**a**) shows the auto-correlation function of realized volatilities for all the time scales. Sub-figure (**b**) presents the probability distribution of *C*_*ij*_ of cross-correlation matrix for each time scale.

The auto-correlation function for each time scale is calculated. Then we take the average over the 225 stocks for each time scale, and the results are displayed in Fig 1(a). After analyzing the auto-correlation function of realized volatilities for all the time scales, we observe that the realized volatility in financial dynamics is also long-range correlated in time, i.e., for each time scale A(t) decays by a power law. A(t) for the high-frequency time scale is more stable and less deviating, while the behavior of the low-frequency is more deviating.

The consistent power-law decay observed across all time scales suggests that the market dynamics have a universal characteristic in terms of volatility persistence.

### Basic characteristics of the cross-correlation matrix

Next, we construct the cross-correlation matrix for each time scale to explore the interdependencies between different stocks. This matrix allows us to quantify the degree of synchronization in price movements, revealing how information spreads across the market. By analyzing the eigenvalues, eigenvectors, and probability distribution of the correlation coefficients, we gain a deeper understanding of the structure and behavior of financial networks.

The probability distribution of *C*_*ij*_, i.e., *P*(*C*_*ij*_) is displayed for each matrix in Fig 1(b). The average value of *C*_*ij*_ is close to 0.27 for the 5-min time scale, 0.25 for the 10-min time scale, 0.22 for the 30-min time scale, 0.21 for the 1-hr time scale, 0.20 for the 2-hr time scale, and 0.18 for the 4-hr time scale. It indicates that the correlation between stocks is larger in the high-frequency time scales, showing that the high-frequency data contain more information about the intra-day movement of the prices.

After that, we calculate the eigenvalues and eigenvectors of the cross-correlation matrix C for each time scale. Following the cross-correlation decomposition, the eigenvalues are computed for the cross-correlation matrix for each time scale. The largest four eigenvalues for each time scale are displayed in Table 1. λ_*max*_ for the 5-min time scale is the largest among the six time scales, which is 62.9, while the λ_*max*_ for the 4-hr time scale is the smallest which is 43.1.

**Table 1:** The values of the largest four eigenvalues for each time scale.

	λ_*max*_	λ_1_	λ_2_	λ_3_
5 min	62.86	9.18	6.75	5.41
10 min	59.46	9.04	6.62	5.36
30 min	51.89	8.49	6.35	5.46
1 h	49.00	8.58	6.10	5.11
2 h	46.51	8.72	6.08	5.20
4 h	43.05	8.94	6.34	5.24

λ_*max*_ represents the largest eigenvalue, λ_1_, λ_2_, λ_3_ are the second, third and the fourth largest eigenvalues, respectively.

It indicates that the stocks are more correlated, and some behaviors of the price movement are only shown in the high-frequency time scale. The results showing the sector mode is more microscopic than the market mode because high-frequency data have more information, allowing for a more detailed distinction between global and local interactions. Therefore, we tend to believe that low-frequency correlation matrices are not as accurate in characterizing interactions as high-frequency data.

The largest four eigenvectors corresponding to the largest four eigenvalues are also obtained for six time scales. We introduce a threshold *U*_*c*_: |*U*_*i*_(λ*α*)| ≥ *U*_*c*_, to select the dominating components in a particular eigenvector. The threshold *U*_*c*_ is set to be 0.08 in this research.

**Fig 2:**
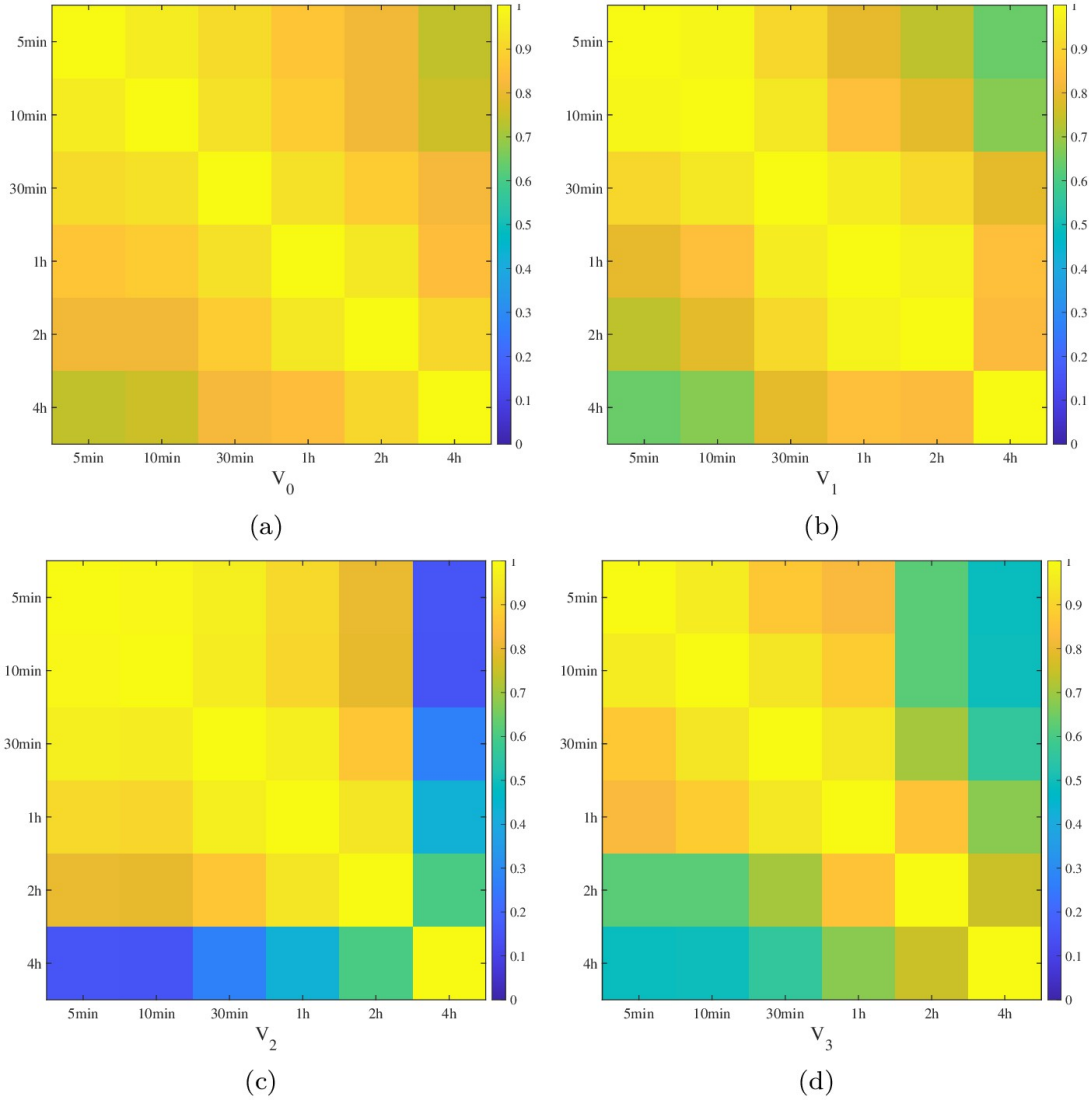
The correlations between the four largest eigenvectors, i.e., *V*_0_, *V*_1_, *V*_2_, and *V*_3_ for the six time scales. Sub-figures (**a**)– (**d**) correspond to *V*_0_, *V*_1_, *V*_2_, and *V*_3_, respectively.

We calculate the correlation between eigenvectors, and the results for the largest four vectors are displayed in Fig 2.

From the results we observe that the correlations between 5-min time scale and 4-hr time scale are smaller than that between other time scales, indicating the behavior of 5-min time scale is different from that of 4-hr time scale.

The analysis of the cross-correlation matrices reveals significant interdependencies between stocks, particularly at shorter time scales. This high degree of correlation at finer time scales highlights the importance of high-frequency data in capturing detailed intra-day price movements. The differences in eigenvectors across time scales illustrate the dynamic nature of market relationships, which has implications for portfolio diversification and systemic risk assessment.

### Community structures across six time scales

**Fig 3:**
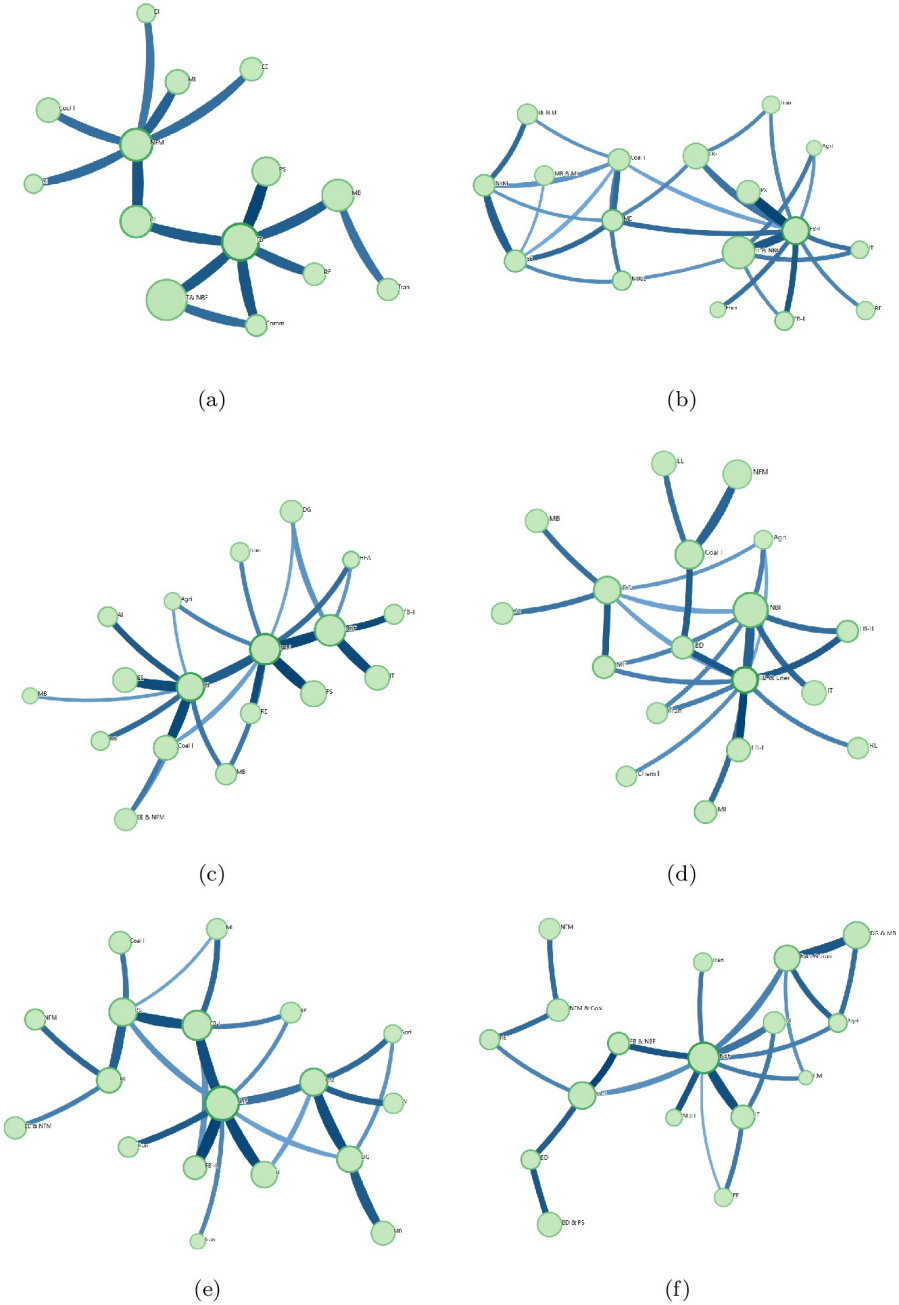
The community structures for different time scales. Sub-figures (**a**)– (**f**) represent the community structures of 5-min, 10-min, 30-min, 1-hr, 2-hr, and 4-hr time scales, respectively. The abbreviations of the business sectors are introduced in the caption of Table 2.

To further understand the structural properties of the financial market, we perform a community structure analysis using the PMFG and Infomap method [44]. Assuming that there are *N* stocks, the solution generates a graph embedded on a surface with a particular genus *g*. The genus of a surface is the number of holes in the surface and *g* = 0 corresponds to a topological sphere, *g* = 1 to a torus, *g* = 2 to a double torus, etc. The graph generated by the PMFG algorithm is a triangulation of the surface and it contains 3*N* + 6*g*—6 links, which maximize the sum of *C*_*ij*_. The simplest case is the graph with *g* = 0 [43]. We first generated PMFG graph from the cross-correlation matrix *C*_*ij*_ of the financial market. Then the Infomap method is applied to capture the interaction structure of the communities from the PMFG graph. This approach helps us identify clusters of stocks that exhibit similar behavior, providing insights into the market’s organization and the role of different sectors. With the PMFG and Infomap method, we investigate the community structures for six time scales, and the results are displayed in Fig 3. The abbreviations of the business sectors are listed in Table 2.

**Table 2:** The names of the business sectors and their corresponding abbreviations.

Abv	Sector Name
IT	Computer
NBF	Non-Bank Financial
FB	Financial Bank
NFM	Non-ferrous Metal
MB	Medical Biology
CI	Chemical Industry
PS	Public Service
MI	Military Industry
Coal I	Coal Industry
EE	Electrical Equipment
RE	Real Estate
Tran	Transportation
Comm	Communication
AI	Automobile Industry
EI	Electronic Industry
DG	Daily Consumer Goods
ME	Mechanical Equipment
BD	Building Decoration
HEA	Household Electric Appliances
FB-I	State-owned bank
FB-II	Joint-stock bank or private bank
Agri	Agriculture
Null	No obvious category

The first column display the abbreviations for the Sector Names. “Null” represents the one we could not identify.

There are 14 business sectors for the 5-min time scale, the most important business sector is IT & NBF, and the other three dominating ones are FB, NFM, and MB. There are 17 sectors for the 10-min time scale, with IT & NBF, FB, DG, and PS being the dominant ones. For the 30-min time scale, there are 18 sectors, and the dominating ones are NBF, FB, Chem I, and PS. For the 1-hr time scale, there are 18 sectors, and the dominating ones are NBF, NFM, Coal I, and DG. For the 2-hr time scale, there are 18 sectors, and the dominating ones are NBF, FB, PS, and CM. For the 4-hr time scale, there are 17 sectors, and the dominating ones are NBF, FB, DG & MB, and Ener & Tran.

From the results, we can observe that the community structure varies for different time scales, and among all the time scales the NBF is a dominating business sector, indicating the Non-Bank Financial sector plays a very important role in the Shanghai and Shenzhen stock markets. Except for NBF, business FB is also a dominating one for all the time scales. As we know, financial bank plays an important role in economic development, promoting capital circulation, risk management, money supply, and innovative development.

**Fig 4:**
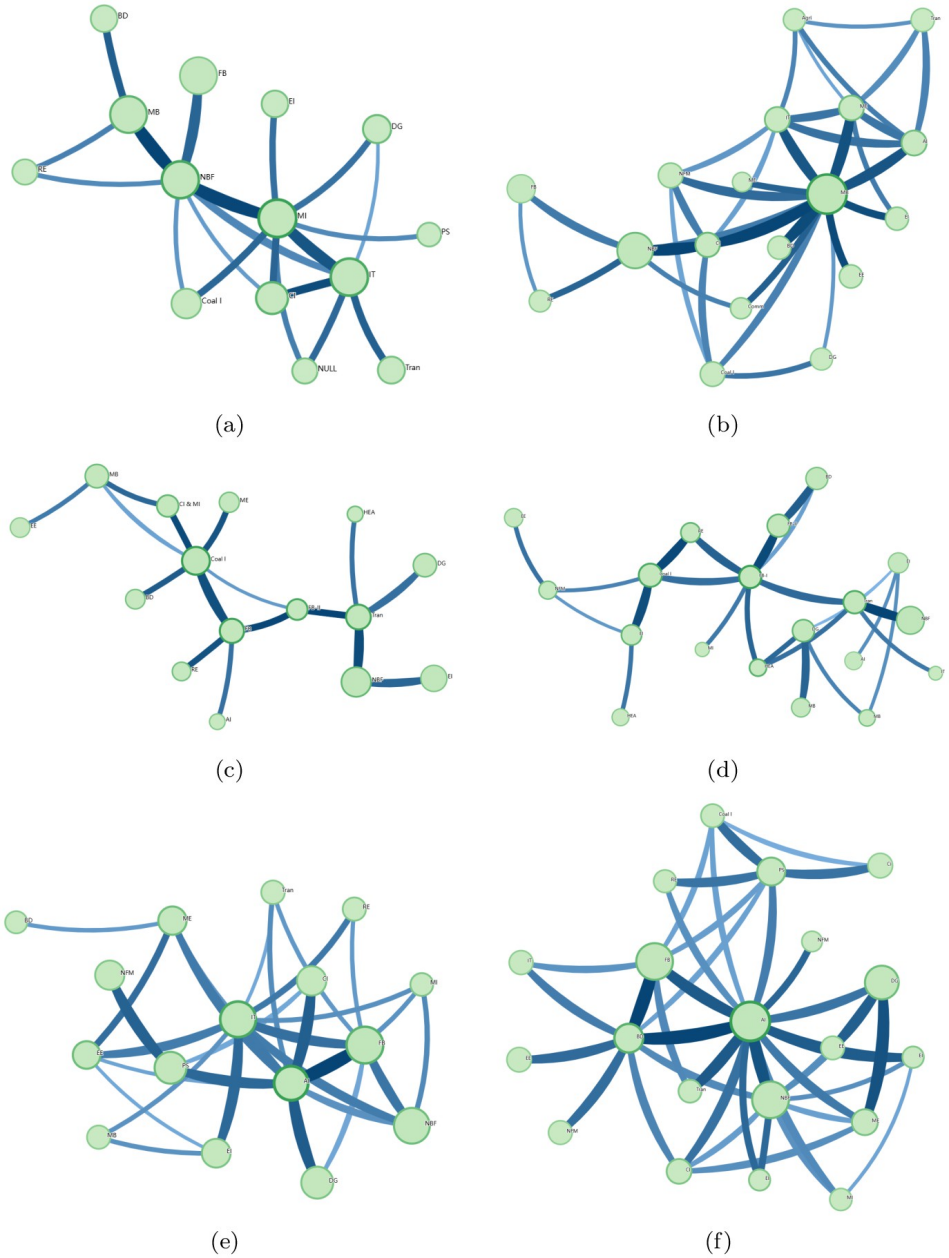
The community structures of 5-min time scale and 4-hr time scale for the three time periods. Sub-figures (**a**)– (**f**) represent the community structures of 5-min and 4-hr time scales for the three time periods. From top to bottom are those for 2016-2018, 2018-2020, and 2020-2022, respectively. Left are those of the 5-min time scale, while right are those of the 4-hr time scale. The abbreviations of the business sectors are introduced in the caption of Table 2.

To test the robustness of the above results, we divide the data into three periods: 2016–2018, 2018–2020, and 2020–2022. The community structures for the 5-min and 4-hr time scales in each period are examined. From Fig 4, we can observe that the community structure of the 4-hr time scale is more complicated than that of the 5-min time scale, which indicates that the community structure for the 5-min scale differs significantly from that of the 4-hr scale in each time period. It is consistent with the former conclusion and confirms the robustness of our findings.

**Fig 5:**
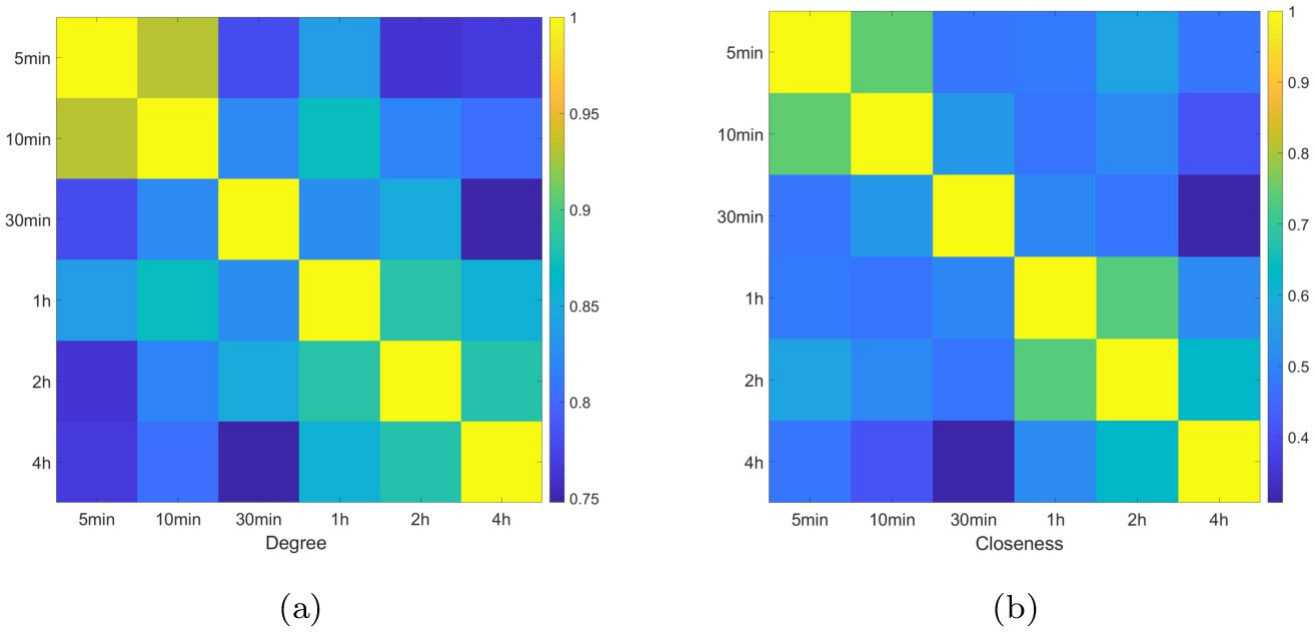
The correlations between the degree and closeness of each node for the six time scales. Sub-figure (**a**) shows the correlations between node degree corresponding to the 5-min and 4-hr time scales, respectively. Sub-figure (**b**) illustrates the correlations between node closeness for the 5-min and 4-hr time scales, respectively.

We further explore how the network’s topological properties, such as node degree and closeness centrality distributions, vary across different time scales [45]. We calculate the correlation between degree and closeness centrality for each node in the PMFG graphs, ranging from the 5-min time scale to 4-hr time scale. The results are presented in Fig 5. The findings indicate that the correlation between the 5-min and 4-hr time scales is lower than that of other time scale pairs, suggesting that the network’s topological properties at the 5-min scale differ significantly from those at the 4-hr scale. This divergence reflects the distinct market dynamics and interaction patterns observed at shorter intervals, where high-frequency trading activity has a more pronounced impact.

The community structure analysis demonstrates that market organization is time-scale dependent, with dominant sectors such as NBF and FB consistently playing key roles. These findings suggest that certain sectors have a stable influence on market dynamics, regardless of the time scale. Understanding these stable community structures can aid in better market segmentation and targeted investment strategies, enhancing overall market efficiency.

### Reaction speed following big events across six time scales

In this part, we first investigate the reaction speed for each time scale average for all stocks after big events. Among the year from 2016 to 2023, we choose two big events to investigate the reaction of the stocks among different time scales. The two events are displayed in Table 3.

**Table 3:** The information about the two big events.

Event 1	2021.1.28	The individual investors “blood washing” Wall Street.
Event 2	2022.4.1	The Shanghai blockade because of the COVID-19.

**Fig 6:**
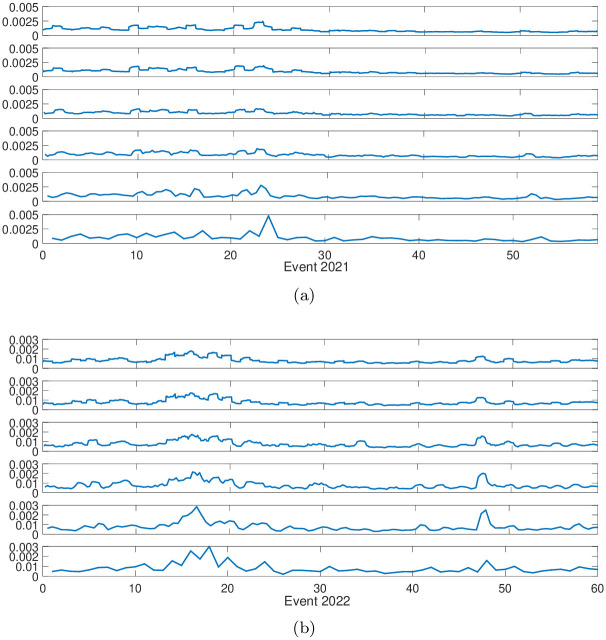
The reaction of stocks across different time scales after big events is illustrated, ranging from the 5-min scale at the top to the 4-hr scale at the bottom. Sub-figure (**a**) corresponds to Event1, while sub-figure (**b**) corresponds to Event2.

Stock markets will be volatile after big events, therefore, for better investigation we compute the realized volatility averaged for 225 stocks for different time scales. The results are displayed in Fig 6. The reaction time (speed) is defined by the time (speed) the first peak of the averaged realized volatility appears. The shorter time indicates a faster reaction speed. With this definition, we investigate the reaction speed for each time scale.

We find that the reaction time for the high-frequency time scale is much earlier than that for the low-frequency. For Event 1, the average volatility increases quickly for the 5-min, 10-min, and 30-min time scales, within less than 1 day. However, for the 4-hr time scale, it decreases after the first day, increases until the fourth day, then decreases and remains stable for three days, and increases again after the seventh day. For the 5-min time scale, the average volatility decreases after two days and becomes stable for three days, while it remains volatile for the low-frequency time scale.

For Event 2, the average volatility for the 5-min time scale increases within one day, also faster than that for the 4-hr time scale. From Fig 6b, we can observe that it decreases on the second day, remains stable for four days, and increases again after the sixth day. For the 4-hr time scale, the average volatility increases after one day. From these observations we conclude that the reaction speed after big events for the high-frequency time scale is much faster than the low-frequency, and it is volatile for the high-frequency time scale while that for the low-frequency time scale is stable, indicating some information only appears or can be observed in the high-frequency time scale. Some research uncovers that minute data can provide price volatility information in a shorter period of time and can quickly respond to market changes [17, 19, 46–48]. In contrast, the daily price data reflects the overall price trend within a trading day. The daily price data is relatively stable because it covers a long period of time and is less affected by short-term factors. This may be why the reaction speed after big events for the high-frequency time scale is much faster than the low-frequency.

After investigating the reaction speed across multiple time scales following big events, we gain a better understanding that high-frequency data contain more information about the price movement, especially the information that cannot observed in the day time scale. It may help to make better investment strategies for the investors, as it may be helpful for getting some risk early warnings and more information about the stock price.

Secondly, we investigate the reaction speed of different industries to major market events across multiple time scales. This analysis helps us understand how quickly different industries respond to significant information, which is crucial for market timing and risk management. By grouping stocks into various industries, we can identify which industries are more sensitive to big events and how their reactions vary across time scales. Following this concept, we group the 225 stocks into eight different industries, i.e., Health care, Electronic industry, Basic materials, Public service, Light industry, Real estate and finance, Energy, and Daily consumer goods. Then, we compare the reaction speed after big events for different time scales of the eight industries.

**Fig 7:**
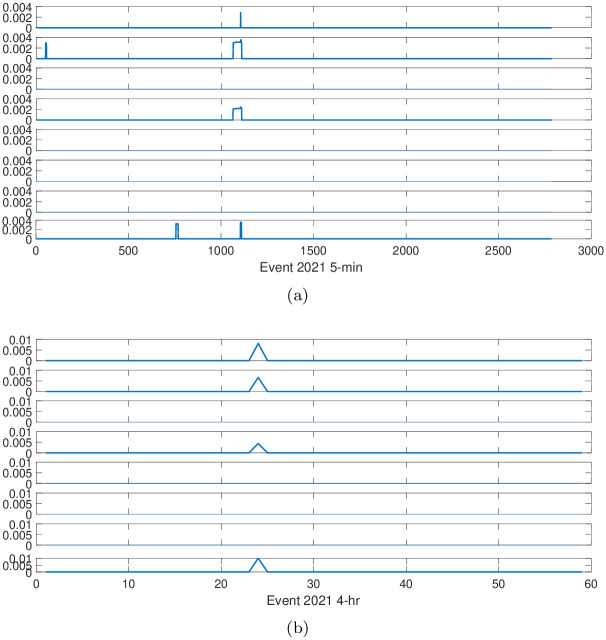
The reactions of different industries to Event 1. Sub-figure (**a**) corresponds to 5-min time scale, while sub-figure (**b**) corresponds to 4-hr time scale. From top to bottom, the industries are Health care, Electronic industry, Basic materials, Public service, Light industry, Real estate and finance, Energy, and Daily consumer goods, respectively.

**Fig 8:**
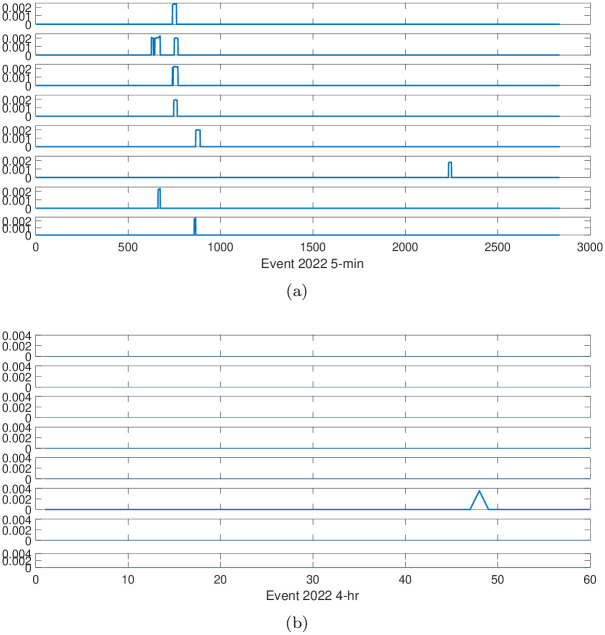
The reactions of different industries to Event 2. Sub-figure (**a**) corresponds to 5-min time scale, while sub-figure (**b**) corresponds to 4-hr time scale. From top to bottom, the industries are Health care, Electronic industry, Basic materials, Public service, Light industry, Real estate and finance, Energy, and Daily consumer goods, respectively.

To obtain a better understanding of the reaction speed for each industry, we introduce a threshold. The threshold is set as 0.8 times its own standard deviation. With this threshold, we investigate the reactions of different industries to big events and compare the difference between time scales, especially for the 5-min scale and 4-hr scale. The results are displayed in Figs 7 and 8. For Figs 7b and 8b we were unable to obtain meaningful information, indicating the data of high-frequency time scale contain more information, which will be helpful for understanding the stock price movement.

So we focus on the behavior of 5-min time scale. From Fig 7a we observe that the Basic materials, Light industry, Real estate and finance, and Energy are stable after the event 2021, while the Health care, Electronic industry, Public service, and Daily consumer goods are more volatile after the event 2021, especially for the Electronic industry and Daily consumer goods. Among these industries, the Basic materials and Light industry are long period, and are less influenced by the event, showing more stability compared to the other industries.

In Fig 8a we observe the Public service, Light industry, Real estate and finance, and Daily consumer goods are stable after the event 2022. The Public service, Light industry, and Daily consumer goods are very close to people’s daily life, and the consumers of these industries are not very sensitive to external events in the market. Among these industries, the Health care, Electronic industry, Basic materials, and Energy are more sensitive to the event.

We all know that after the year 2020, COVID-19 swept the world and affected all aspects of human society, such as life and health, and population mobility. This may be the reason that the Health care, Electronic industry, Public service, Basic materials, and Energy are more volatile after the event 2022.

The faster reaction speed observed at high-frequency scales indicate that certain sectors quickly assimilate and respond to significant market events. Identifying these responsive industries can provide valuable insights for investors seeking to capitalize on short-term opportunities and for policymakers aiming to enhance market stability. These findings underscore the importance of high-frequency data in capturing timely market reactions and informing strategic decisions.

The Volatility Impulse Response Function (VIRF) simulates how financial market volatility responds to shocks over time [49]. It measures the effects of a one-time shock across various time horizons, revealing the duration, magnitude, and speed of market reactions. The process involves estimating a volatility model, like a GARCH model, using historical data to describe volatility behavior without shocks. Consider a GARCH(1,1) model defined as

$$\begin{array}{c} r_{t}=\mu +\epsilon_{t}, \end{array}$$ (9)

$$\begin{array}{c} \epsilon_{t}=\sigma_{t}z_{t}, \end{array}$$ (10)

where *r*_*t*_ represents asset returns, *μ* is the mean return, *ϵ*_*t*_ is the error term, *z*_*t*_ is a white noise process (usually assumed to be normally distributed), and the conditional variance $\sigma_{t}^{2}$ is modeled as

$$\begin{array}{c} \sigma_{t}^{2}=\alpha_{0}+\alpha_{1}\epsilon_{t-1}^{2}+\beta_{1}\sigma_{t-1}^{2}. \end{array}$$ (11)

Here *α*_0_, *α*_1_, and *β*_1_ are model parameters to be estimated based on the historical returns. To analyze the impact of a shock, assume a one standard deviation shock occurs at time *t* = 0 (e.g., *ϵ*_0_ = *σ*), where *σ* represents the standard deviation of the returns *r*_*t*_. This shock is intended to model a significant event, such as a policy announcement. The conditional variance at this time can be expressed as

$$\begin{array}{c} \sigma_{t}^{2}{|}_{t=0}=\alpha_{0}+\alpha_{1}\cdot \sigma^{2}+\beta_{1}\cdot \sigma_{t-1}^{2}. \end{array}$$ (12)

The VIRF quantifies the response of volatility

$$\begin{array}{c} \text{VIRF}\left(h\right)=E\left[\sigma_{t+h}^{2}|\epsilon_{0}=\right]\sigma -E\left[\sigma_{t+h}^{2}|\epsilon_{0}=0\right]. \end{array}$$ (13)

Here, we define *r*_*t*_ as *R*_*i*_(*t*′, Δ*t*) to simulate the VIRF respectively and take the average across all stocks for each time period. The time lag *h* ranges from 1 to 60 days.

**Fig 9:**
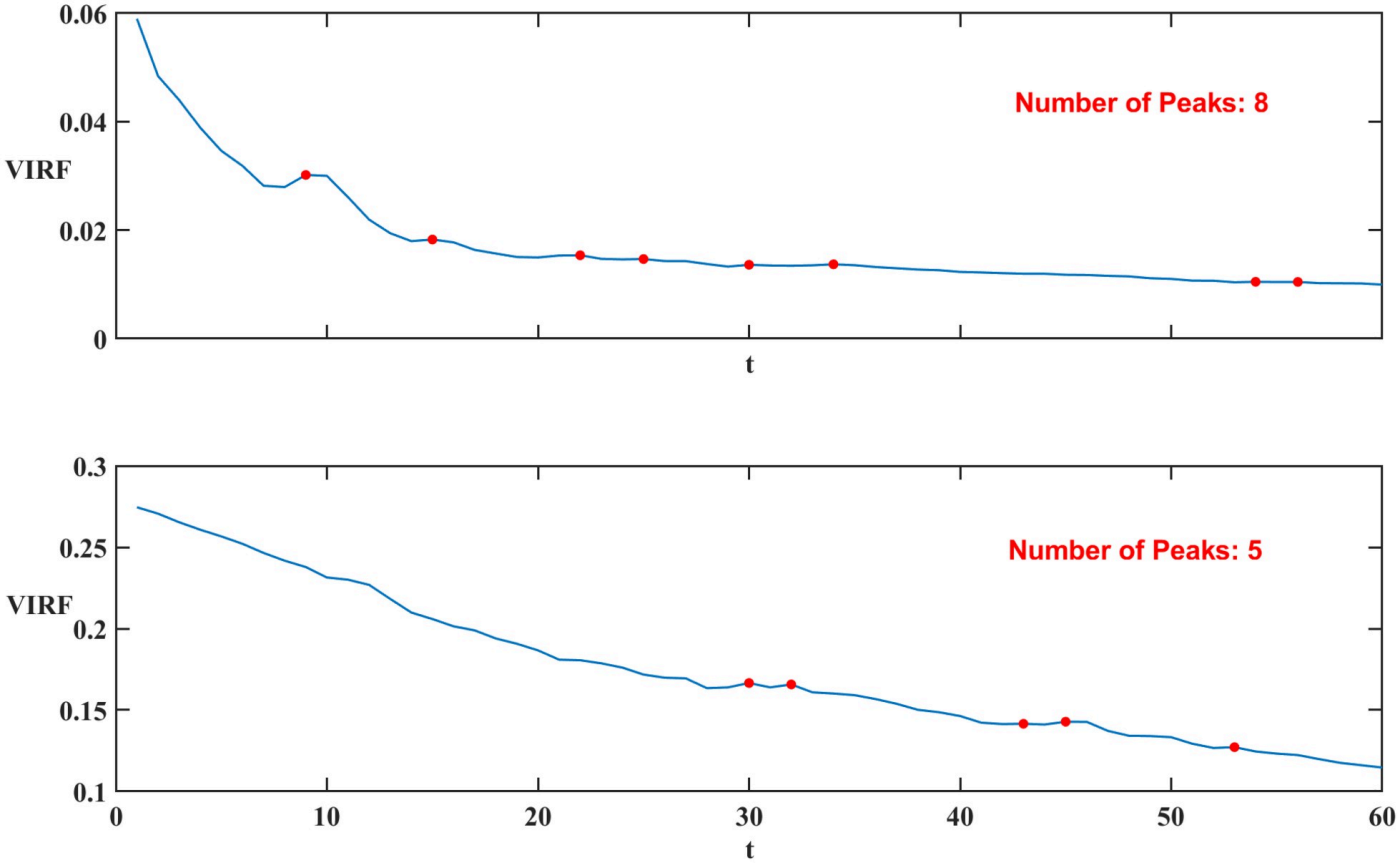
VIRFs for Event 1 corresponding to 5-min time scale (up) and 4-hr time scale (bottom) respectively.

To explore the dynamics of the market’s reaction to significant events, we simulate the VIRFs for the volatility following Event 2021 across various time scales, ranging from 5-min time scale to 4-hr time scale. As illustrated in Fig 9, the results reveal that the 5-min time scale exhibits more peaks, with the initial peak occurring significantly earlier than the 4-hr time scale. Other time scales also consistently exhibit this pattern. The results for Event 2022 are also similar. This observation is consistent with our empirical findings, suggesting that markets operating at higher frequencies are more sensitive and respond more rapidly to shocks. In other words, time series data at shorter intervals not only exhibit higher volatility but also indicate a faster reaction speed when absorbing and adjusting to new information, compared to longer, low-frequency intervals. This underscores the importance of analyzing market dynamics across multiple time scales to fully understand the temporal characteristics of volatility and market behavior.

## Conclusion

Based on the 5-min high-frequency data of China’s financial institutions from 2016 to 2023, we have obtained the realized volatility for each stock for six time scales, i.e., 5-min, 10-min, 30-min, 1-hr, 2-hr and 4-hr. The auto-correlation function of the realized volatility for each time scale is calculated, showing a similar power-law decay across all time scales. Then we construct the cross-correlation matrix for each time scale, the eigenvalues, eigenvectors, and the probability distribution of *C*_*ij*_ are investigated. The correlation between stocks is larger in the high-frequency time scales, indicating that the high-frequency data contain more detailed information about intra-day price movements. Additionally, the eigenvectors corresponding to large eigenvalues differ across time scales.

Afterward, with the PMFG method, the community structures across six time scales are analyzed. We observe that the community structure varies for different time scales. Notably, the Non-Bank Financial (NBF) sector consistently dominates, highlighting its significant role in the Shanghai and Shenzhen stock markets. Alongside NBF, the Financial Bank (FB) sector also remains prominent across all time scales, underscoring its crucial role in economic development, capital circulation, risk management, money supply, and innovation.

Furthermore, the reaction speed across multiple time scales following big events is investigated. Our analysis reveals that high-frequency time scales react much faster than low-frequency scales. By categorizing stocks into eight industries and examining their reactions to major events, especially at the 5-min time scale, we identified which industries are more sensitive to these events. The results of this part may help to make better investment strategies for the investors, as the high-frequency data contain more information about the stock price movement, it may be helpful for getting some risk early warnings for the financial policymakers and regulators in policy-making, regulations design, portfolio management, risk management, and trading.

These findings not only enhance our understanding of the complex dynamics of financial markets but also provide practical applications. High-frequency data analysis offers a more precise tool for predicting market volatility and improving market stability. Future research should further explore the application of high-frequency data under diverse market conditions and integrate multiple data sources for comprehensive market analysis. This will provide richer references and support for financial market research and practical applications.

This study faces some challenges, particularly related to the use of high-frequency data intervals, which can capture intra-day market dynamics but may also introduce noise from market microstructure effects, such as bid-ask spreads and transaction costs. Future research could expand on this work by analyzing international markets for comparative volatility dynamics and investigating the effects of external macroeconomic variables or news sentiment on high-frequency market behavior.

## Abbreviations

10-min: 10-minute
1-hr: 1-hour
2-hr: 2-hour
30-min: 30-minute
4-hr: 4-hour
5-min: 5-minute
HFT: High-frequency trading
PMFG: Planar maximally filtered graph
RMT: Random Matrix Theory
